# Twenty years of surveillance in Rett syndrome: what does this tell us?

**DOI:** 10.1186/1750-1172-9-87

**Published:** 2014-06-19

**Authors:** Alison Anderson, Kingsley Wong, Peter Jacoby, Jenny Downs, Helen Leonard

**Affiliations:** 1 Telethon Kids Institute, The University of Western Australia, PO Box 855, West Perth, WA 6872, Australia; 2 School of Physiotherapy and Exercise Science, Curtin University, GPO Box U1987, Perth, WA 6845, Australia

**Keywords:** ‘Rett syndrome’, Survival, Ageing, Longevity, Adulthood, Wellbeing, Women

## Abstract

**Background:**

The clinical characteristics of children diagnosed with Rett syndrome are well described. Survival and how these characteristics persist or change in adulthood are less well documented. This study aimed to describe overall survival and adult health in those with Rett syndrome.

**Methods:**

Using the Kaplan-Meier method, we estimated survival of individuals registered with the Australian Rett syndrome Database (ARSD) who had been followed for up to 20 years (n = 396). We then conducted logistic and linear regression analyses investigating epilepsy, musculoskeletal, gastrointestinal, autonomic dysfunction and behaviour of individuals aged 18 years and over using cross sectional cohorts from the ARSD (n = 150) and the international database InterRett (n = 273).

**Results:**

The likelihood of survival was 77.6% at 20 years, 71.5% at 25 years and 59.8% at 37 years. The median age of the combined cross-sectional cohort was 25 years (range 18 to 54 years), the majority (71%) were living in their parental home and the remainder being cared for in group homes or other institutions. Just over half walked either independently (18%) or with assistance (43%). The majority (86%) had scoliosis with 40% of those having undergone corrective surgery. Almost two-thirds (64%) of the women were taking anti-epileptic medications at the time of data collection. Constipation was highly prevalent (83%) and many experienced bloating (53%). Biliary dyskinesia, inflammation or infection of the gallbladder was reported for 20 women (5%) and of those 13 had undergone gallbladder surgery. Sleep disturbance was relatively common (63%), and adverse mood events and anxiety were slightly more prevalent in those aged 26-30 years in comparison to the younger and older age groups. Other frequently reported medical conditions included urinary tract infections, pneumonia and other respiratory conditions.

**Conclusions:**

Survival in Rett syndrome has now been estimated with the most accurate follow up to date. During adulthood, continuation of multidisciplinary services and programs is necessary to optimise health and wellbeing.

## Background

The neurological disorder Rett syndrome (OMIM 312750) was first described in the English literature in 1983 [1] and later found to be associated with mutations in the methyl CpG binding protein 2 gene (*MECP2*) [2]. Females are mostly affected with an incidence of diagnosis of 1:9000 by the age of 32 years [3]. Diagnosis depends on clinical presentation [4] with or without a pathogenic *MECP2* mutation. Genetic testing occurs commonly in current diagnostic pathways but many older women may not have undergone genetic testing. The clinical characteristics of Rett syndrome first appear in early childhood. Gradual or sudden loss of speech and hand function, loss of acquired gross motor skills and the development of stereotypic hand movements mark a period of regression between the ages of 6 and 18 months. Gastrointestinal problems, respiratory dysfunction such as hyperventilation, breath holding and apnoea, sleep disturbance, spinal curvature and epilepsy are common comorbidities. Overall severity of symptoms is highly variable across individuals and studies have identified common mutations associated with either a milder phenotype (point mutations p.Arg133Cys, p.Arg294*, and p.Arg306Cys) or a more severe clinical presentation (point mutations p.Thr158Met, p.Arg168*, p.Arg255*, p.Arg270* and large deletions) [5,6]. Clinical presentation in adults with Rett syndrome is less well understood.

### Emerging picture of health and wellbeing in adults with Rett syndrome

Thus far only two studies conducted in the Netherlands (2007: n = 53, 2012: n = 37) [7] and Italy (n = 84) [8] have specifically examined health in adults with Rett syndrome. Overall, general health was reported to be good. However epilepsy was found to be highly prevalent in both the Dutch (93%) and the Italian cohorts (82%), and while improvement in seizure activity with age was noted for some women [8], some remained difficult to treat and the majority required medications for control of seizures. A post-adolescence decline in gross motor skills has been reported in some [7,9] but not all women with Rett syndrome [7]. Gross motor capabilities are influenced by the type of mutation present in the *MECP2* gene and are generally poorer in those requiring surgical correction for scoliosis [10]. Autonomic dysfunction which may manifest as hyperventilation or breath holding, or peripheral vasomotor disturbances is a well-recognised but poorly understood feature of Rett syndrome. Abnormal or disturbed sleep patterns and behavioural issues also appeared to persist [7,8]. Similarly, gastrointestinal issues such as constipation, reflux, and feeding difficulties remained prevalent [7,8] and may contribute to poor growth [11]. However, there is a need for replication of these studies using larger sample sizes to improve our understanding of clinical presentations beyond the growth and development of childhood.

### Survival

It is possible that those adults with Rett syndrome who survive into adulthood may represent a healthier group. The most recent Australian population-based estimate indicated that ~70% would be alive at 25 years with some evidence of improved survival over calendar time when comparing with the historical Austrian cohort [12]. A study using data on 1,907 cases ascertained from a combined US and Canadian sample (n = 2,994) showed a similar result at age 25 years [13]. A recent longitudinal study conducted in The Netherlands [7] reported the death of 7 of 53 women aged between 21 and 43 years over a five-year period. However, it is difficult to measure survival accurately using small samples that are not population-based with only short follow-up time.

Our aims were to update our previous estimate of survival with now 20 years of follow-up in the Australian Rett Syndrome Database (ARSD) and to describe health status in individuals aged 18 years or older sourced from both the ARSD and the InterRett database.

## Methods

### Data sources

Data for this study were harnessed from two data repositories: The ARSD and the international database InterRett. The ARSD is a population-based registry established in 1993 [14] representing individuals born since 1976. Questionnaires are administered on enrolment to both families and clinicians and subsequently, follow-up questionnaires are administered approximately biennially to families. The InterRett database was first established in 2002 and invites participation from both families and clinicians on a global level [15]. Families who register are administered a family questionnaire and one of the clinicians caring for their family member with Rett syndrome is also invited to complete a clinician questionnaire. Ethics approval for both studies was obtained from the Princess Margaret Hospital for Children, Western Australia.

### Survival

Female cases with a confirmed diagnosis of Rett syndrome registered with the ARSD (n = 396) were included. Data on deceased status and cause of death (excluding cases that were subject to a coroner’s investigation) were obtained from the Australian Institute of Health and Welfare (AIHW) National Death Index (NDI) database and were valid as at 31st January 2014. Age at first contact with the ARSD was used as entry points and age at death or age at last contact date if alive was used as exit points. Time as risk was defined as the difference between the entry and exit points, reflecting the delayed entry nature of the study. As such, the interpretation of the survival time is that of survival to exit age conditional on survival up to the age at entry. The censor status of each case was set as 1 if the individual was deceased and 0 if the individual was known to be alive.

### Health status

Females aged 18 years or older at their most recent data collection were sourced from both the ARSD (n = 150) and InterRett (n = 273) databases providing a total of 423 unique cases. Rett syndrome diagnosis was confirmed at ascertainment based on consensus diagnostic criteria [4,16,17] or on the presence of a pathogenic mutation in the *MECP2* gene.

Demographic information and variables relevant to health status that had been harmoniously ascertained across the two databases were selected for analysis. Clinical comorbidities included epilepsy, musculoskeletal aspects (mobility, scoliosis), gastrointestinal health (weight, constipation, reflux, bloating and gall bladder problems), autonomic dysfunction, sleep disturbance, and mood and anxiety. Where more detailed information on a particular health area was available in one data source but not the other, this information was analysed and reported for the relevant subset of the cohort. Families had also been asked to describe any medical problems (current or present) other than those usually associated with Rett syndrome or that had required day admissions or surgery in hospital. This information was reviewed to determine whether or not any particular medical problems were more represented.

Ability to walk was categorised as one of the following: no assistance required, a little assistance (eg, one hand held), a moderate amount of assistance (eg, needing trunk support) or unable to walk. Epilepsy status was categorised according to frequency of seizures and the number of anti-epileptic medications. Active epilepsy represents those with seizure frequency of at least monthly at the time the questionnaire was completed, and drug resistant epilepsy was defined as having active epilepsy and taking two or more anti-epileptic drugs at the time of data collection [18].

Responses to the Rett Syndrome Behaviour Questionnaire (RSBQ) [19], which has been administered for the Australian cohort, were used to calculate scores for the mood and anxiety subscales. The mood subscale comprised eight questions describing behaviours such as screaming spells and irritability with 3 response options (0-2) giving a possible total score of 16. The anxiety subscale score comprised four questions describing behaviours such as panic spells and fear with 3 options (0-2) giving a possible total score of 8. Higher scores indicated more problematic behaviours.

Also for the Australian cohort, weight z-scores for age were calculated using the LMS method based on data from the 2000 US Centers for Disease Control and Prevention Growth [20]. The reference value of age 20 years was applied to any individual over this age.

Mutations were grouped as large deletions (LD), C-terminal deletions (CT), early truncating (ET) or one of the common eight mutations. All others were grouped as “other” mutations. Females who were mutation negative or with unknown mutation status were included in the “Negative/Unknown” (Neg/Unk) group. Age was grouped as 25 years and younger, between 26 and 30 years, and older than 30 years for health status comparisons.

### Statistical analysis

The Kaplan-Meier method was used for estimating the conditional probability of survival. Cox proportional hazards models were fitted to investigate the influence of genotype on survival and logistic regression was used to estimate the associations of mutation type and age with the presence of comorbidity. The p.Arg133Cys group, a mutation associated with an overall milder phenotype [21], was used as a baseline. The effects of mutation type on continuous independent variables such as weight, RSBQ mood score and RSBQ anxiety score were assessed using simple linear regression. Predicted values of these explanatory variables were then estimated based on the recycled predictions approach using the Stata *margins* command.

All statistical analyses were conducted using Stata software version 13 [22].

## Results

### Survival

**Figure 1:**
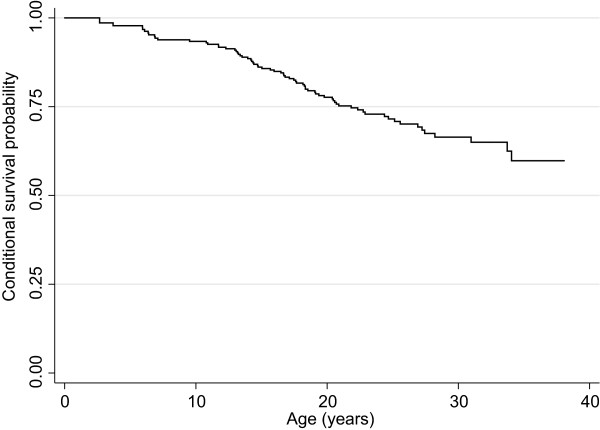
Kaplan Meier survival curve for 396 girls and women with Rett syndrome.

**Figure 2:**
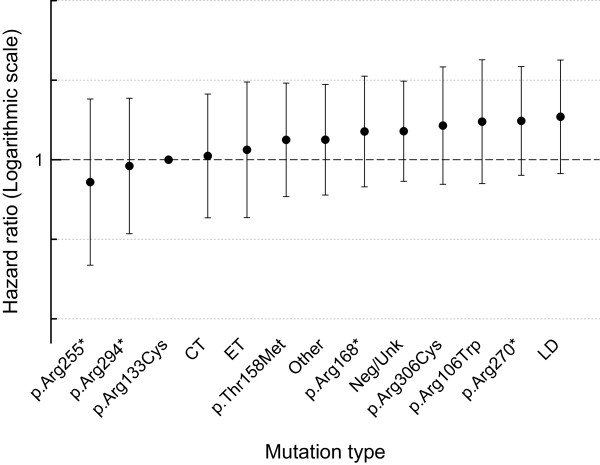
**Risk of death during the observation period in the Australian cohort (n = 396) by common mutation groups in comparison to the p.Arg133Cys mutation group.** The error bars denote 95% confidence intervals. CT, C-terminal deletion; ET, early truncating; LD, large deletion; Neg/Unk, mutation negative or unknown.

As in January 2014 the ARSD contained birth and death information for 396 females (median age 18.3, inter-quartile range (IQR) 12.1-25.4 years). Information on cause of death was available for 57 (82.6%) out of 69 females who were deceased. Lower respiratory tract infection (36.8% 21/57) was the most common cause of death, followed by aspiration/asphyxiation (31.6% 18/57), respiratory failure (14.0% 8/57) and seizure related illness (5.3% 3/57). The conditional probability of survival was 77.6% (95% confidence interval (CI) 71.3, 82.8) at 20 years, 71.5% (95% CI 64.6, 77.3) at 25 years and 59.8% (95% CI 49.3,68.8) at 37 years (Figure 1). There were 300 females in the ARSD with a pathogenic mutation in the *MECP2* gene (Table 1) and 96 females with negative *MECP2* testing or unknown genotype. Using the Cox proportional hazard model, those with large deletions had, at any time during the observation period, slightly more than 3 times (HR 3.11, 95% CI 0.60, 16.10) the risk of death than those with the p.Arg133Cys mutation. High mortality risk was also observed in those with p.Arg270* (HR 3.05, 95% CI 0.63, 14.73), p.Arg106Trp (HR 2.24, 95% CI 0.45, 11.11) and p.Arg306Cys mutations (HR 2.66, 95% CI 0.49, 14.53) (Figure 2). The risk was lowest for those with the p.Arg255* mutation (HR 0.52, 95% CI 0.05, 5.76).

**Table 1:** Number (%) of girls and women in the survival analysis for each mutation type (n = 300)

**Mutation type, n (%)**	
C-terminal	27 (9.0)
Early truncating	21 (7.0)
Large deletion	21 (7.0)
p.Arg106Trp	14 (4.7)
p.Arg133Cys	23 (7.7)
p.Arg168*^a^	34 (11.3)
p.Arg255*	18 (6.0)
p.Arg270*	25 (8.3)
p.Arg294*	24 (8.0)
p.Arg306Cys	19 (6.3)
p.Thr158Met	32 (10.7)
Other	42 (14.0)

^a^^*^stop codon.

### Health status

The combined study population represented 24 countries with the greatest numbers residing in the USA (40.4%, 171/423), Australia (38.3%, 162/423), Canada (5.9%, 25/423) and the UK (5.7%, 24/423). Age at the time of data collection ranged from 18.0 to 54.3 years (median 24.9 years, IQR 21.5-30.7 years). Over two thirds of the women (71.2%, 301/423) were living in their parental home or with other family members and 29% (122/423) were being cared for in facilities such as group homes and other institutions. The characteristics of the women are shown in Table 2.

**Table 2:** Number (%) for each mutation type and medical condition in the health status analyses (n = 423)

	**ARSD**^**a **^**(n = 150)**	**InterRett (n = 273)**	**Overall (N = 423)**
**Mutation type, n (%)**			
C-terminal	11 (9.9)	8 (6.6)	19 (8.2)
Early truncating	7 (6.3)	6 (4.9)	13 (5.6)
Large deletion	6 (5.4)	5 (4.1)	11 (4.7)
p.Arg106Trp	4 (3.6)	6 (4.9)	10 (4.3)
p.Arg133Cys	10 (9.0)	7 (5.7)	17 (7.3)
p.Arg168*^c^	12 (10.8)	9 (7.4)	21 (9.0)
p.Arg255*	9 (8.1)	10 (8.2)	19 (8.2)
p.Arg270*	9 (8.1)	8 (6.6)	17 (7.3)
p.Arg294*	9 (8.1)	7 (5.7)	16 (6.9)
p.Arg306Cys	6 (5.4)	6 (4.9)	12 (5.2)
p.Thr158Met	12 (10.8)	4 (3.3)	16 (6.9)
Other	16 (14.4)	46 (37.7)	62 (26.6)
Total	111^d^	122^e^	233
**Medical conditions, n/N**^**b **^**(%)**			
Seizures	64/144 (44.4)	122/267 (45.7)	186/411 (45.3)
Unable to walk	70/150 (46.7)	97/272 (35.7)	167/422 (39.6)
Scoliosis	122/150 (81.3)	237/270 (87.8)	359/420 (85.5)
Constipation	111/146 (76.0)	221/255 (86.7)	332/401 (82.8)
Bloating	85/147 (57.8)	121/244 (49.6)	206/391 (52.7)
Gallbladder problems	3/144 (2.1)	17/233 (7.3)	20/377 (5.3)
Gastro-oesophageal reflux diseases	22/144 (15.3)	-	22/144 (15.3)
Gastrostomy	41/150 (27.3)	-	41/150 (27.3)
Altered breathing patterns	97/146 (66.4)	-	97/146 (66.4)
Sleep disturbances	92/142 (64.8)	154/249 (61.9)	246/391 (62.9)

^a^Australian Rett Syndrome Study; ^b^the denominator is different for each medical condition because of some families did not compete all questionnaire sections; ^c^*stop codon; ^d^negative or unknown mutation (n = 39); ^e^negative or unknown mutation (n = 151).

### Epilepsy

**Figure 3:**
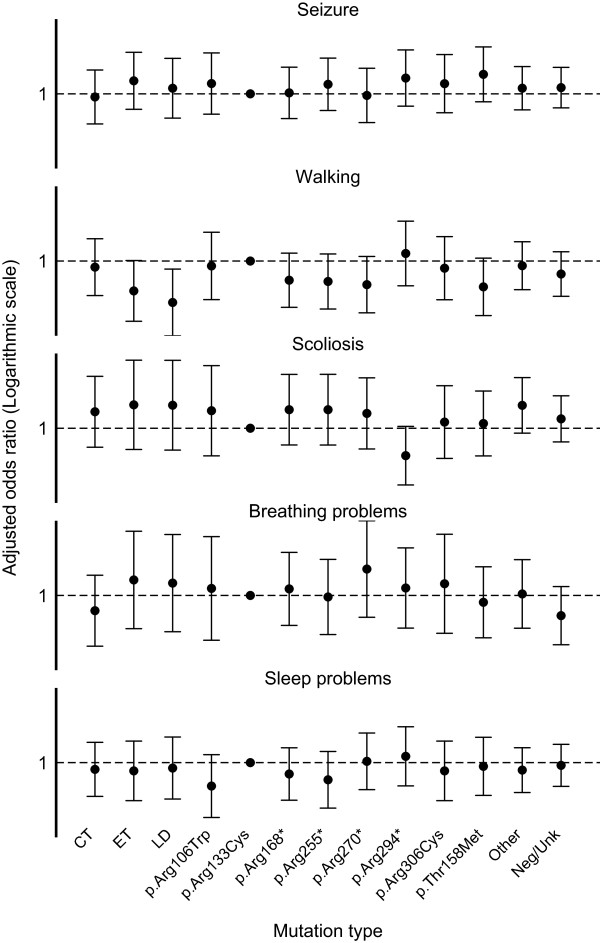
**Influence of mutation type, adjusted for age, on selected health conditions.** The error bars denote 95% confidence intervals. Seizure (n = 411) was defined as having active epilepsy. Walking (n = 422) was defined as able to walk with or without assistance. Scoliosis (n = 420) was defined as ever had scoliosis. Breathing problems (n = 146) were defined as having a breathing problem including breath holding and hyperventilation. Sleep problems (n = 391) were defined as having sleep disturbances. CT, C-terminal deletion; ET, early truncating; LD, large deletion; Neg/Unk, mutation negative or unknown.

Almost two-thirds (64.0%, 263/411) of the women were taking anti-epileptic medications at the time of data collection with only 17.3% (71/411) having never experienced seizures. Of those receiving anti-epileptic medication, seizures were completely controlled in 38.0% (100/263), partially controlled in 25.9% (68/263), and were classified as drug resistant in 36.1% (95/263). A small proportion (5.6%, 23/411) was not taking anti-epileptic medication despite some level of seizure activity and 13.1% (54/411) who had previously been diagnosed with epilepsy, were currently seizure free. Some families reported a reduction in severity or the number of seizures with increasing age while others reported increased seizure activity. The p.Thr158Met mutation group had the highest odds of individuals having active epilepsy (OR 2.77, 95% CI 0.66, 11.67) (Figure 3). There were no significant differences in prevalence of active epilepsy across mutation types.

### Musculoskeletal aspects

Many of the women continued to walk independently (17.8%, 75/422) or with a little (22.5%, 95/422) or moderate amounts of assistance (20.1%, 85/422). Slightly fewer than half (39.6%, 167/422) were unable to walk at all at the time of data collection. Those with a large deletion (OR 0.11, 95% CI 0.02, 0.65), or a mutation that resulted in early truncation of the protein (e.g. p.Gly269fs) (OR 0.21, 95% CI 0.04, 1.03) appeared to be more severely affected when compared to those with the p.Arg133Cys mutation (Figure 3).

### Scoliosis

Scoliosis had developed in the majority (85.5% 359/420). The proportion of women who could not walk was greater in those with scoliosis (45.3%, 162/358), in comparison to those with no spinal curvature (8.2%, 5/61). Across mutation types the odds of having scoliosis was lowest in women with the p.Arg294* mutation (OR 0.24, 95% CI 0.05, 1.10) and highest with a large deletion (OR 3.34, 95% CI 0.32, 35.00) (Figure 3). Information on scoliosis treatment was provided by 337 of the families whose daughter had scoliosis and in 40.0% (135/337) had undergone spinal fusion, mostly between the ages of 11 and 16 years.

### Growth and gastrointestinal disorders

**Figure 4:**
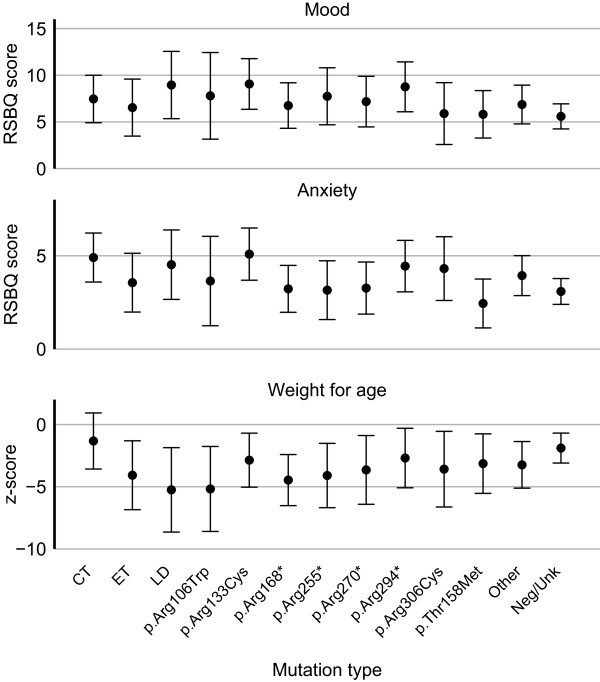
**Age and mutation type adjusted predicted scores of mood and anxiety and estimated weight-for-age z-scores.** The error bars denote 95% confidence intervals. The RSBQ mood score ranges from 0 to 16 (n = 137) and the anxiety score ranges from 0 to 8 (n = 137). Higher scores represent higher frequency of adverse behavioural events. CT, C-terminal deletion; ET, early truncating; LD, large deletion; Neg/Unk, mutation negative or unknown.

Information on body weight, method of feeding and presence of reflux was only available for cases ascertained from the ARSD. The mean weight-for-age z-score was -3.04 (SD 3.39). The majority of women (52.8%, 65/123) were underweight (z-score ≤ -2.0) including some (8/65) who were markedly underweight (z-score ≤ -10). Slightly fewer than half (46.3%, 57/123) were in the normal weight range and one was overweight (z-score ≥ 2.0). Weight-for-age z-scores were lowest in those with large deletions (Predicted value -5.25, 95% CI -8.64, -1.86) and those with p.Arg106Trp (Predicted value -5.18, 95% CI -8.59, -1.77) mutations and highest in those with C-terminal mutations (Predicted value -1.32, 95% CI -3.58, 0.93) (Figure 4).

Also for the Australian cohort, 27.3% (41/150) had had a gastrostomy feeding tube inserted to: improve food/fluid intake; prevent aspiration; or to provide relief from bloating. Almost half (46.2%, 18/39) of those receiving nutrition through gastrostomy also had some oral intake, 15.4% (6/39) had all food orally and 38.5% (15/39) had all food/fluid intake by gastrostomy. Four women had had a gastrostomy for a short period during childhood and were now feeding orally.

Reflux was reported for 15.3% (22/144) of women in the Australian cohort. For the combined Australian and InterRett cohorts, constipation was highly prevalent (82.8%, 332/401), and many were experiencing bloating (52.7%, 206/391). Biliary dyskinesia, inflammation or infection of the gallbladder was reported for 20 women (5.3%, 20/377) and of these 13 had undergone gallbladder surgery.

### Breathing patterns

Information on breathing patterns was available for the Australian cohort. Abnormal breathing patterns were reported for two thirds of women (66.4%, 97/146) including 74.2% (72/97) who hyperventilated and 88.7% (86/97) with breath-holding or apnoeic episodes. Within this group, 62.9% (61/97) both hyperventilated and breath-held. These breathing patterns varied little across mutation types (Figure 3).

### Sleep disturbances, mood and anxiety

Sleep disturbances were common (62.9%, 246/391) and almost half the women (47.7%, 178/373) experienced night laughing. Night screaming occurred often for 13.2% (18/136), occasionally for 20.6% (28/136) and not at all for 66.2% (90/136) of ARSD women. In comparison to p.Arg133Cys, sleep disturbance was more prevalent in those with p.Arg294* (OR 1.39, 95% CI 0.30, 6.55) and less common in those with p.Arg106Trp mutations (OR 0.29, 95% CI 0.06, 1.52) and those with p.Arg255* (OR 0.41 95% CI 0.09, 1.79) (Figure 3).

Families participating in the InterRett study reported that 46.0% (109/237) experienced screaming spells during the day. Otherwise, information on mood and anxiety was only available for those in the ARSD (n = 137). Information pertinent to mood and behaviour, obtained using the RSBQ, indicated that adverse behaviour such as screaming spells and irritability occurred often (RSBQ score >=11) in 19% of women, occasionally (RSBQ score 6-10) in 41% and rarely (RSBQ score <=5) in the remainder (40%). Anxiety, as determined by response to four related RSBQ questions was experienced often (RSBQ score >=7) for 11%, occasionally (RSBQ score 3-6) for 58% and rarely if ever (RSBQ score <=2) for 31%.The average score for mood was 6.86 (SD 4.07) out of a possible total of 16. After adjusting for age group, predicted mood scores above the average (indicating difficult behaviour) were observed for mutation groups considered to have a milder phenotype, p.Arg133Cys (Predicted score 9.07, 95% CI 6.36, 11.78) and p.Arg294* (Predicted score 8.76, 95% CI 6.09, 11.44) and also those with a large deletion (Predicted score 8.95, 95% CI 5.35, 12.56) who generally have a more severe overall phenotype (Figure 4). Individuals within the 26-30 years age group were likely to have a slightly higher mood scores (Predicted score 8.54, 95% CI 7.00, 10.09) compared to younger women (Predicted score 6.50, 95% CI 5.59, 7.41) and those over 30 years of age (Predicted score 6.16, 95% CI 4.60, 7.72) after adjusting for mutation type.The average score for anxiety was 3.66 (SD 2.14) out of a possible total of 8. In comparison to those with p.Arg133Cys mutations (Predicted score 5.09, 95% CI 3.69, 6.48), women with p.Thr158Met had reduced anxiety (Predicted score 2.45, 95% CI 1.14, 3.76) as did those with p.Arg168* mutations (Predicted score 3.23, 95% CI 1.97, 4.49) after adjusting for age group (Figure 4). As with mood, those aged 26-30 years were likely to experience slightly more episodes of anxious behaviour (Predicted score 4.21, 95% CI 3.41.5.01) compared to younger women (Predicted score 3.62, 95% CI 3.15, 4.08) or those over 30 years (Predicted score 3.20, 95% CI 2.40, 4.01) after adjusting for mutation type.

### Other health issues

Responses to questions regarding hospitalisations, investigations and treatments for health issues other than those normally associated with Rett syndrome revealed 11 conditions reported for at least 4 individuals (Table 3). Of these, urinary tract infections and respiratory conditions, including pneumonia, were the most commonly reported.

**Table 3:** Other health conditions reported (n = 320)

**Medical condition, n (%)**	
Pneumonia	55 (17.2)
Urinary tract infection, pyelonephritis, bladder infection	48 (15.0)
Ear infection	36 (11.2)
Tonsillitis	23 (7.2)
Asthma	20 (6.2)
Bronchitis	19 (5.9)
Respiratory illness	13 (4.1)
Heel cord, foot surgery	12 (3.7)
Kidney stone	5 (1.6)
Insulin resistance	5 (1.6)
Polycystic ovaries/ovarian cyst	4 (1.3)

## Discussion

Using a population-based cohort we have shown that current survival to the age of 20 years is 77.6% with 59.8% surviving to early middle age. In women aged 18 years and older we found that epilepsy, breathing and sleep problems persisted, low weight and gastrointestinal issues were prevalent and although most have scoliosis loss of walking is not inevitable. The influence of genotype, while moderate, is in agreement with previous findings.

A major strength of this study is the rich data sources available for analyses. Our population-based ARSD with follow-up of individuals up to 20 years was coupled with up-to-date death information has allowed us to make an accurate estimate of survival in Rett syndrome. Our cross-sectional analysis of a large multi-country study population, over half of whom have a confirmed mutation in the *MECP2* gene has allowed us to more fully investigate the clinical profile, and influence of genotype, in ageing women. Limitations of this study include lack of representation of older women not living in the parental home in the international community since, unlike those who live in their parental home, their family members would be less likely to be active participants in online initiatives such as the InterRett database. Despite the survival analysis being undertaken on Australian population-based data collected over 20 years the capacity to detect differences in mortality risk across mutation groups was limited by the small number of deaths within specific mutation groups.

Importantly, we can now report survival in the ARSD cohort of slightly less than 60% at 38 years of age although the current estimate of survival to 25 years (71.5%) is lower compared to our 2006 analysis (77.8%) [23]. Death was most commonly caused by respiratory illnesses. Increased ascertainment and follow-up of individuals in the more recent analyses likely explains the difference in the survival estimate over time. A higher estimate of survival to 25 years (~80%) was reported for a cohort of US and Canadian females (n = 1,907) [13]. The authors of this study reported that many known individuals who had been identified through the family association were not included in their analysis due to failure to make contact (52%, n = 1,555) and it is likely that individuals who have not registered with the family association or clinics also exist within the US and Canadian populations. The survival estimate is likely over-estimated because it is quite plausible that the proportion of deceased compared to living cases would have been higher in the group whose families were not able to be contacted in comparison to the group whose families were able to be contacted and on whose data the analysis was based. As recommended by the authors of the study, a population-based national registry, similar to that in Australia, with links to national death registry and other pertinent data sources might be advantageous, as mortality in such cohort will yield more precise survival rates as case numbers and periods of follow-up increase over time.

Compared to those with the p.Arg133Cys mutation, the risk of death was high in those with a p.Arg270* mutation consistent with our previous findings [24]. Our new findings indicate that survival in those with the p.Arg106Trp mutation or large deletion is similar to that of the p.Arg270* group and that those with p.Arg255* mutations demonstrate better survival. A proportion of those who were older had not been tested for a *MECP2* mutation because of its relative recent availability and some families do not now want to revisit diagnostic processes. Nevertheless, genetic testing for women with a clinical diagnosis of Rett syndrome has important roles to play. Some families do value more specific diagnostic information [25] and the data contributes to greater accuracy in future estimations of the effects of genotype on phenotype including survival.

Our cross-sectional analyses used the largest sample size to date for investigating health and wellbeing in adulthood. Seizures are common in Rett syndrome and not surprisingly, almost two-thirds (263/411) of the women continued to take anti-epileptic medications at the time of data collection. Previous studies have reported stabilisation or improvement in epilepsy in older women [7]. While some parents in the current study noted that the frequency or severity of seizures had diminished with age, the majority had active seizures, and of these, a third was considered to be refractory. An investigation of epilepsy in the InterRett study showed similar results for those aged 17 years or older [18]. These findings indicate that management of epilepsy remains a serious challenge in ageing women and this picture was consistent across all mutation types.

Over half (60%) of the study cohort had some walking skills at the time of data collection. The proportions of women who could walk independently, with assistance, or not at all, were similar to that previously reported for an Italian cohort of women and adolescents aged 14 or more years [8]. A longitudinal study of adult women with Rett syndrome conducted in the Netherlands [7] indicated that age-related deterioration of gross motor function is slow. This observation concurs with findings from a video study which investigated general stability in gross motor function over a 3- to 4-year period: girls younger than 13 years with walking skills were more likely than teenagers and women to lose the more complex motor skills that enabled better negotiation of the environment [26]. Together these findings add strength to the thesis that loss of walking is not inevitable as women with Rett syndrome age. It is likely, however, that these women represent the milder range of the severity spectrum and that those who could not walk from an early age, being more severely affected, may have died before reaching adolescence or adulthood. Those with spinal curvature were less likely to be ambulant and encouragingly, many were well following previous surgical management.

The proportion of those who could walk in the p.Arg133Cys mutation baseline group (76%) was similar to those with a p.Arg294* mutation (81%) and significantly better than those with large deletions (27%). Improved gross motor skills for those with a p.Arg133Cys and p.Arg294* has been previously observed in a video study of 99 girls and women from the ARSD registry, 29 of whom were 19 years or older, possibly reflecting some survival bias [10]. A 2008 study (n = 272) found that the overall phenotype of those with p.Arg133Cys and p.Arg294* mutations is mild [5] and Cuddapah et al [6] found significant differences in ambulation, not adjusted for age, between those with p.Arg133Cys and nine other mutation groups (p.Thr158Met, p.Arg168*, p.Arg255*, p.Arg270*, Deletions, Insertions, Large Deletions, Splice Sites and No mutations). These consistent findings across and within age groups help to provide a clearer clinical picture of mobility outcomes in those affected by these common mutations.

We found, as had others, that sleep disturbance [7,8] and gastrointestinal problems [7] were highly prevalent in older women. We recently found the rate of gall bladder disease in a Rett syndrome population-based study to be 2.3 per 1000 person years (ie. two new cases if 100 individuals are followed for 10 years) [27]. Available population-based data on typical paediatric populations suggested a lower prevalence than this [28,29]. Although gall bladder disease was identified in 5% of adults in our current study it is unclear how this compares with the general population. We suggest that gall bladder disease should be considered in the differential diagnosis of abdominal pain in Rett syndrome.

Unusual breathing patterns including hyperventilation and breath holding were highly prevalent yet there has been a paucity of research in this area. Women with a p.Arg255* mutation were least likely to hyperventilate and also had a lower mortality. The influence of breathing irregularities on overall health and survival is not well understood and further research in this area is warranted. Similarly, the frequency of respiratory type infections indicates that these health issues may also need to be closely monitored, including attention to upright postures during daytime activities and a low threshold to the prescription of antibiotics.

Fortunately, behavioural difficulties and anxiety only appeared to affect a relatively small proportion of women although these issues would likely have a major impact on the daily quality of life of those with Rett syndrome and their carers. Small increases in the mood and anxiety scores were observed for those aged 26-30 years in comparison to the older and younger age groups. In the Dutch cohort, behaviour was assessed using the Observational Questionnaire Elderly Residents with Intellectual Disabilities (OOB). Based on this instrument, two thirds of Dutch women showed anxiety and during the five year study period improvements were mainly seen in those age 30 years or more (2007-2012) [7]. It might be expected that those with a milder phenotype can better communicate their feelings, however, we observed higher predicted scores for mood and anxiety in mutations associated with both mild and severe phenotypes.

The change in composite health status by mutation type over time was outside the scope of the current study. However, a previous study of health trajectories over time in our Australian cohort [30] found that for most mutations, including p.Arg133Cys where health status at a younger age was better, health status deteriorated over time. For a few others, which tended to be severe mutations, health status either remained unchanged (pArg106Trp) or improved slightly (p.Arg255* and p.Arg168*) with age [30]. The authors of the US longitudinal study [6] reported that severity of symptoms increased by age for most mutation types in both cross-sectional analysis across five age groups and longitudinal analysis within individuals. The severity score for an individual was determined by a protocol developed by the authors who acknowledged that the inclusion of some items, which cannot change with time, may have led to an underestimation of age-related changes in their longitudinal analyses. Given that there is considerable variance in clinical outcomes within mutation types, consistent ongoing follow-up of existing cohorts is required to obtain a clearer picture of mutation-specific severity in women with Rett syndrome.

## Conclusion

The data contained in this paper represents twenty years of surveillance and highlights the consistent research effort required to understand rare disorders. Individuals with Rett syndrome have potential for prolonged survival with approximately 60% surviving to early middle age. Many women continue to have some level of ambulation and it is therefore critical to develop and advocate for care plans that help to maintain and build on these skills. Further investigation into mood and anxiety could help to better define needs and inform care strategies. We also recommend that the maintenance of clinical care services for adults with Rett syndrome including close monitoring of respiratory and gastrointestinal function. The impact of breathing irregularities on survival is poorly understood and is an important topic for future investigation. Ongoing monitoring of the ARSD and other population-based cohorts has a vital role in providing accurate estimates of survival in Rett syndrome.

## Competing interests

The authors declare that they have no competing interests.

## Authors’ contributions

The study was conceptualised by HL and JD both of whom participated in the organisation and execution of the study including drafting and critique of the manuscript. AA participated in the study design, organisation and statistical analysis and wrote the first draft of the manuscript. KW and PJ designed and executed statistical analysis and all authors contributed to the drafting and critique of the manuscript. All authors read and approved the final manuscript.
